# Transport pathways across the blood-brain barrier for waste clearance and drug delivery

**DOI:** 10.1186/s12987-026-00812-7

**Published:** 2026-05-16

**Authors:** Lea S. Gröbner, Claus U. Pietrzik

**Affiliations:** 1 Institute for Pathobiochemistry, University Medical Center, Mainz, Germany; 2 Molecular Neurodegeneration, Institute for Pathobiochemistry, University Medical Center, Mainz, Germany

**Keywords:** Blood-brain barrier, Neurovascular unit, Transcytosis, Waste clearance, LRP1, TfR1, Drug delivery, Antibody modification, Nanocarrier, PCSK9 inhibitors

## Abstract

The blood-brain barrier (BBB) displays a highly organized and complex structure, which is important for maintaining brain homeostasis and protecting the brain from foreign molecules or pathogens. Receptor-mediated transcytosis (RMT) is one of the main delivery pathways across the BBB for molecules that cannot pass the barrier via, e.g. paracellular diffusion. For understanding the treatment options in neurodegenerative diseases such as Alzheimer´s disease (AD), it is important to investigate transport pathways and mechanisms at the BBB for a potential delivery of drugs, antibodies or other compounds across the BBB. This review provides an overview of the different transport variants across the BBB and how they can be targeted in order to promote internalization or secretion into or out of the brain. Therefore, we want to focus on two characterized proteins: the low-density lipoprotein receptor-related protein 1 (LRP1), which is a key mediator of amyloid β (Aβ) clearance from the brain during AD, and transferrin receptor 1 (TfR1), which is already used as a target for antibody-delivery into the brain. Additionally, this review discusses two other important proteins, which have been less frequently addressed in research regarding transport mechanisms: P-glycoprotein (P-gp) as another transporter at the BBB and proprotein convertase subtilisin/kexin type 9 (PCSK9), a well-known regulator of cholesterol homeostasis which promotes the degradation of the low-density lipoprotein receptor (LDLR) and LRP1. For these four main proteins, we aim to highlight existing approaches for targeting or inhibiting the aforementioned receptors or proteins. The approaches enable a higher penetration of the BBB, a better distribution in the brain, and ultimately fewer side effects of antibodies or nanoparticles. Here, we include lecanemab, trontinemab, dual TfR/CD98hc shuttles, evolocumab and alirocumab, immunoliposomes and other nanoparticles targeting TfR1 or LRP1. We will further highlight approaches which differ from these common ideas and demonstrate the current state of the art regarding drug delivery and waste clearance across the BBB.

## Background

The human brain is a highly privileged organ which is complex in organization and signaling for maintaining its physiological function. Within the human brain, many different cell types contribute to the structure and function of the brain parenchyma. These include endothelial cells, astrocytes, and pericytes. Embedded in the basal membrane, those cells form the neurovascular unit (NVU) [1]. The assembly of the NVU is fundamental for delivery of substrates, either into the brain or out of the brain, and efficient intracellular signaling [2, 3]. A disruption of this system can be one common co-incidence of age or neurodegenerative disease, resulting in neuroinflammation or neurological decline, subsequently leading to a cognitive or a motor deficit [4–6]. To circumvent such pathologies, a clearance system across the blood-brain barrier (BBB) or the blood-cerebrospinal fluid (CSF) barrier helps to remove waste products out of the brain and, especially the BBB, regulates the uptake of substrates into the brain parenchyma [7, 8]. Thus, the unspecific internalization of molecules into the brain is restricted to only a few molecules. For most of them, specific channels, transporters, or receptors at the endothelial cell surface at the BBB manage the specific uptake and transcytosis. Namely, the two main proteins, which are also in the focus for drug transport, are transferrin receptor 1 (TfR1) and low-density lipoprotein receptor-related protein 1 (LRP1). TfR1 is important for a luminal (blood-facing) to an abluminal (brain-facing) transcytosis of iron-loaded transferrin (Tf) across the endothelial layer of the BBB [9]. LRP1, in contrast, plays an important role in amyloid β (Aβ) clearance from the brain into the blood [10].

In this review, we will highlight the complexity of the BBB and its overcoming through TfR1 or LRP1-facilitated receptor-mediated transcytosis (RMT) and how these principles can be used to help drugs enter the brain parenchyma. The review will discuss different approaches of drug delivery across the BBB, including: (1) ultrasound-based BBB opening; (2) functionalized antibodies; (3) nanocarrier as liposomes or solid-lipid nanoparticles (SLNs); (4) antisense oligonucleotides (ASOs); (5) viruses; (6) proprotein convertase subtilisin/kexin type 9 (PCSK9) inhibitors. All these approaches aim to facilitate BBB crossing of drugs and antibodies, which subsequently unfold their effect in the brain parenchyma, but the mechanism of action differs.

Antibody functionalization by the modification of the heavy chain leads to a higher affinity to distinct receptors at the BBB, facilitating their internalization by RMT and their release into the brain. Engineering of the light chains can further target different proteins in the brain, e.g. Aβ or beta-site amyloid precursor protein cleaving enzyme 1 (BACE1), resulting in a reduction of Aβ plaques in Alzheimer´s disease mice [11, 12]. Packaging of drugs or compounds into functionalized nanocarriers like liposomes, SLNs, or exosomes, which are able to bind and further be internalized by receptors at the endothelial cells, improves transport across the BBB and enables the substrate to act in the brain [13–17]. Besides directly targeting receptors at the BBB, the expression of the receptors can also be regulated. By using ASOs, the protein expression can be regulated at the RNA level [18]. Last, PCKS9 inhibitors were shown to increase LRP1 expression, thus leading to a higher Aβ clearance rate [19, 20].

Until now, most of these approaches are tested in vivo in mouse or monkey models. Some of the modified antibodies are already used in clinical trials, revealing the effectiveness of this method of treatment. The goal of all these recent approaches is to increase the BBB permeability and therefore to reduce the required dose of the drug. Additionally, the focus lies on the reduction of side-effects such as Amyloid-Related Imaging Abnormalities (ARIA) compared to previously standard therapies [21, 22].

In the following, we will provide insights into the mechanisms of transcytosis at the BBB and list the current state of research on drug delivery across the BBB.

## Barriers in the brain

**Fig. 1:**
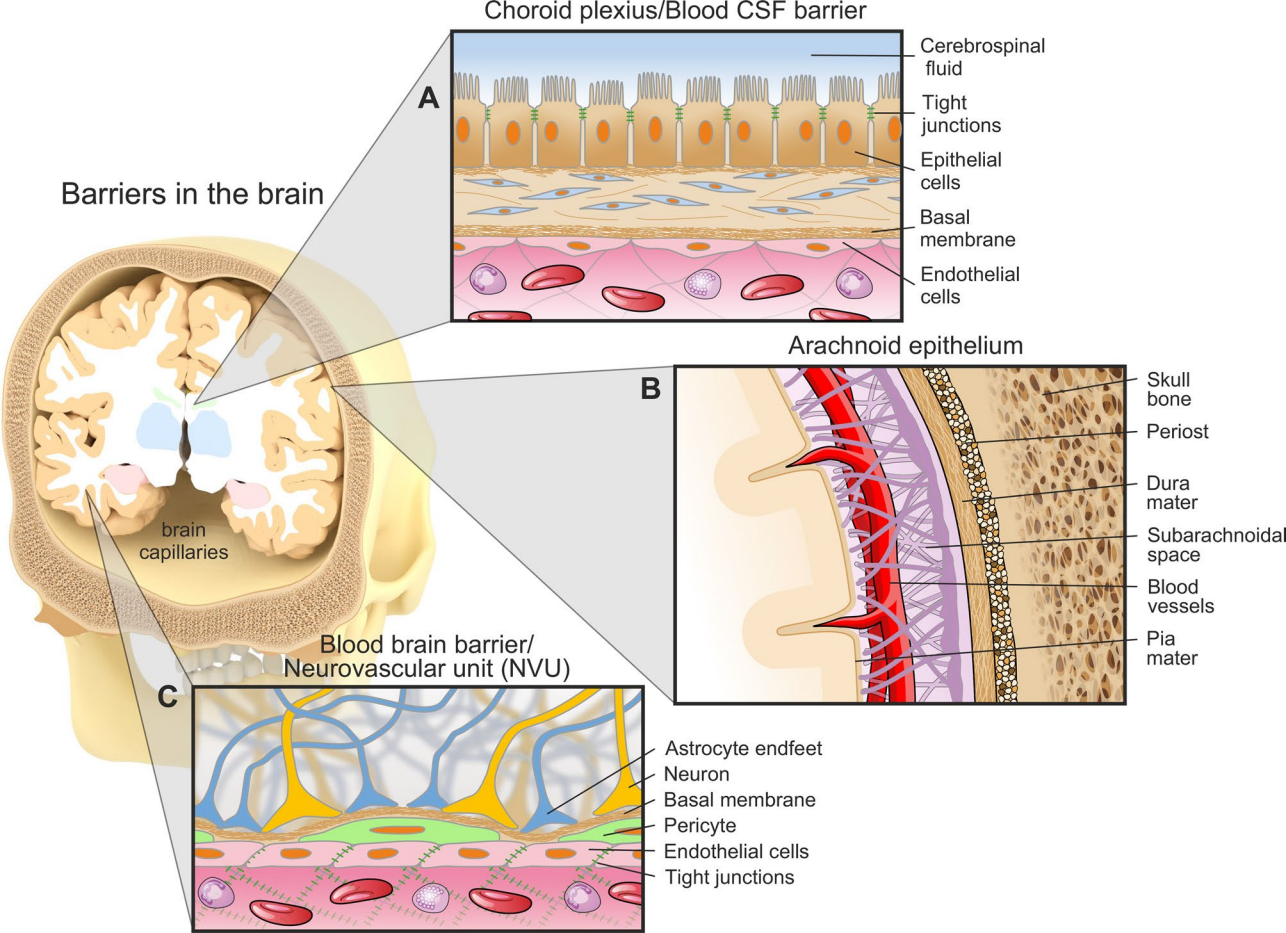
Barriers in the brain. (**A**) The choroid plexus, separating the blood and the CSF. This blood-CSF barrier is mainly responsible for waste clearance from the brain into the periphery. (**B**) The arachnoid epithelium encloses the whole CNS. Due to its vessel-lacking phenotype, this barrier serves rather as a protective barrier than as a surface for exchanging molecules. (**C**) The BBB is the important exchange area in the brain for molecules. Consisting of various cell types shaping the neurovascular unit (NVU), these selective properties are closely regulated

The human body consists of multiple protective barriers that serve to protect essential organs, of which the brain is among the most vulnerable. Although it accounts for only 2% of the total body mass, about 20% of the total oxygen and 25% of glucose levels in the body are consumed by the brain [23–25]. This demonstrates the high need of supply for the brain, which has to be covered by a complex barrier system, preventing pathogens from entering the brain while enabling an exchange of nutrients between the brain and the circulatory system. Paul Ehrlich (1885) and Edwin Goldmann (1919) discovered another barrier between the cerebrospinal fluid (CSF) and the circulatory system [26, 27]. Today, three major barriers in the brain are known: The choroid plexus, shielding the blood and the ventricular CSF; the arachnoid epithelium between blood and the subarachnoid CSF; and the blood-brain barrier (BBB), formed by brain endothelial cells (ECs) and microvessels [28, 29] (Fig. 1).

All three interfaces serve as different kinds of barriers, ranging from physical barriers to transport barriers and metabolic barriers. Through ion channels at the membranes, a homeostasis of ions and pH can be guaranteed [30]. The regulation of neurotransmission is assured through a controlled transcytosis of blood-derived neuroexcitatory amino acids or molecules like glutamate into the brain [31]. Macromolecules in the blood, like pro-thrombin and plasminogen, which can damage neuronal tissue by causing seizures, glial activation, or scarring and even cell death, are kept outside the brain [32]. Additionally, neurotoxins can be retained in the blood by those barriers. That is why maintenance of these barriers is extremely critical and a disruption may result in brain damage, which can become prematurely debilitating [33].

The first barrier, the choroid plexus, is responsible for the secretion of CSF and interstitial fluid (ISF) to the ventricular system of the brain (Fig. 1A). Thus, this barrier is also called the blood-CSF barrier [34, 35]. The second barrier, the avascular arachnoid epithelium, completely encloses the central nervous system (CNS) [36] (Fig. 1B). But because of its blood-vessel-lacking structure and the relatively small area, the arachnoid epithelium does not serve for the exchange of molecules or cells between the blood and the brain [37]. In contrast, the BBB with its exchange area between the microvessels and brain endothelium accounts for the largest barrier with an average surface area of 12 to 18 m [2, 38] (Fig. 1C). It is a highly dynamic and organized structure, regulating the exchange of molecules, ions and cells between the blood vessel and the brain tissue [39]. Breakdown of the BBB is often associated with neurological diseases such as Alzheimer´s disease (AD) [40], Parkinson´s disease (PD) [41], or amyotrophic lateral sclerosis (ALS) [42]. As a consequence, waste clearance or nutrient supply of the brain are impaired and neuroinflammation or neural degeneration can occur. That is why the exact arrangement and maintenance of these barriers is crucial for supporting the physiological function of the brain.

## Arrangement of the blood-brain barrier – the neurovascular unit

The organization of the BBB is based on the network of many different cell types, forming the neurovascular unit (NVU). These include a monolayer of ECs, astrocyte end-feet, pericytes (PCs) and the basal membrane (BM). One main function of the NVU is the maintenance of BBB integrity and brain homeostasis to preserve normal function and health of the brain [1]. The NVU and the BBB composition slightly differs between different brain regions. In rat brains, capillary density was 3–5 times higher in gray matter compared to white matter [43]. Going deeper into the cellular composition of the BBB, it becomes clear that every cell type involved in the formation of the BBB has a distinct role in the arrangement of the NVU.

Astrocytes are star-shaped cells, which are an important component of establishing the BBB. They are the most abundant and diverse cell type of the NVU, covering the whole endothelium-pericyte network with their end-feet processes [44]. Astrocytes also contribute to waste clearance via the glymphatic system thanks to the high expression of aquaporin-4 (AQP4) water channels and potassium channels [45]. Additionally, they secrete factors like Wnt and Norrin factors [46], sonic hedgehog (*Shh*), angiopoietin-1 (Ang-1), insulin-like growth factor (IGF-1), retinoic acid (RA), apolipoprotein E (apoE), and glial-derived neurotrophic factor (GDNF), which regulate the signaling for maintaining a healthy EC pattern and subsequently the formation of the BBB [47–49]. While astrocytes are important for BBB formation, they cannot establish the BBB phenotype on their own. Co-culture studies showed that astrocytes combined with neural or nonneural tissues were insufficient for BBB formation, whereas inclusion of ECs enabled BBB development [50]. Further, astrocytes have a sensory and regulatory function in neurosynaptic transmission and blood vessel function by releasing ATP, glutamate, and D-serine [44, 51, 52].

The BM, a special extracellular matrix surrounding the ECs, serves as another barrier for molecules, which must be transversed to overcome the BBB. It is divided into two different BMs: The inner endothelial BM embeds the endothelial cells and pericytes and consists of laminin alpha4 and alpha5 [53]. The outer parenchymal BM embeds astrocytes and links the astrocyte endfeet to the BM. This BM contains more laminin alpha1 and alpha2 [54]. In both BMs, fibronectin and collagen IV are present for embedment of the other cell types.

PCs are embedded in the BM, surrounding the endothelium at the abluminal site via synaptic-like socket focal contacts through N-cadherin and connexins [55, 56]. Those cells contribute to the BBB maintenance, angiogenesis and vessel stabilization via the expression of catecholamines, angiotensin I, vasoactive intestinal peptides, endothelin-1, and vasopressin [57–62]. Thanks to their close proximity to ECs, ions, metabolites, and signaling molecules can be exchanged between these cells [56]. Further, PCs can recruit immune cells and can react to neuroinflammation themselves by phagocytosing infectious cells or presenting antibodies to immune cells [63].

ECs are flat, polarized cells, forming a tight surrounding of the blood vessels, connected via tight junction (TJ) proteins [8]. Because of the polarized characteristics and the TJs, ECs are the most important cells at the BBB for transcellular transport of molecules and cells between the blood-facing (luminal) site and the brain-facing (abluminal) site [2]. They restrict the paracellular diffusion of macromolecules and other substances but allow oxygen from the blood to diffuse into the brain and carbon dioxide out of the brain. Small lipid-soluble molecules up to 600 Da with less than eight hydrogen bonds are able to cross the barrier passively. Glucose, amino acids, insulin, transferrin, and other important nutrients are dependent on special transporters and carriers, regulating the homeostatic composition in the brain [64].

The closure of the barrier, resulting in a measurable transendothelial electrical resistance (TEER) as a value for the electrical resistance across the endothelial cell layer, is the result of the expression and connection of the TJ proteins between the ECs. In vivo, the TEER value for physiologically tightened ECs is between 1,500–2,000 Ω*cm [2, 65]. The TJs consist of three major integral membrane proteins: claudin, occludin and junctional adhesion molecules (JAM). All of them enable to limit the permeability of the barrier and therefore contribute to maintenance of the brain homeostasis [66].

Claudins are 22 kDa phosphoproteins. They contain four transmembrane domains by which they bind other claudins or cytoplasmatic proteins such as zonula occludens proteins ZO-1, ZO-2 or ZO-3 with their carboxy terminus [67, 68]. The most common forms are claudin-1 and claudin-5 [69]. Occludin is a 65 kDa phosphoprotein which has four transmembrane domains, a long C-terminal cytoplasmic domain and a short N-terminal cytoplasmic domain [68]. Occludin forms paracellular junctions with claudin from the neighboring cells. JAMs are 40 kDa immunoglobulins with a single transmembrane domain and two immunoglobulin-like loops in the extracellular part [68]. JAM-1 and JAM-3 are expressed in the brain blood vessels and are involved in cell-to-cell adhesion and monocyte transmigration through the BBB [70, 71]. Occludin and JAM can interact with cytoplasmic ZO-1 to tighten the barrier [72, 73].

Besides these TJs, adherens junctions (AJs) contribute to the tightening of the BBB. The membrane protein cadherin binds to the actin cytoskeleton in the cytoplasm via intermediate proteins called catenins. For this, cadherin binds to β- or γ-catenin, which are linked to actin via α-catenin [68]. AJs are important for structural maintenance of the cells and the tight junctions [74]. The tightness between the various cell components favors a constricted signaling between them, shaping the signal pathway in the brain.

## Intra- and intercellular signaling for building the BBB

The tight and distinct assembly of ECs, PCs, and astrocytes at the BBB forms the establishment of a homeostatic regulation between the cells themselves, the blood, and the brain. The intra- and intercellular signaling is mainly driven by many different small regulatory proteins. Wnt7a/7b ligands and the Wnt/β-catenin pathway were shown to be a major driver for angiogenesis and barriergenesis [3]. During embryogenesis, neural precursor cells secrete vascular endothelial growth factor (VEGF), leading to angiogenesis through the VEGF/VEGFR pathway [75]. Additionally, Notch signaling leads to a differentiation of these precursor cells, which then secrete the molecule Delta-like 4, managing a coordination of the vascular network [76]. Barrier properties are established through signals from the neuroectoderm by Wnt-, *Shh*-, and transforming growth factor-β (TGF-β) pathways, resulting in EC differentiation [3]. The interplay between those different signaling pathways mediates the distinct assembly and functionality of the brain and subsequently the barriers in the brain.

The canonical Wnt/β-catenin pathway is a central mechanism for the establishment of the BBB. Wnt ligands, such as Wnt7a and Wnt7b, are released from glial and neuronal cells. These bind Frizzled receptors in the presence of LRP5/6, leading to β-catenin stabilization. Subsequently, β-catenin acts as a co-transcription factor with lymphoid enhancer factor/T-cell factor for the regulation of the cell cycle, apoptosis, cell differentiation, and cellular communication [77]. For the BBB, this Wnt/β-catenin pathway leads to the transcriptional activation of e.g. TJ proteins or transporters, shaping the BBB structure [78]. Another pathway is the hedgehog pathway through *Shh,* which induces protein kinase A and/or G-protein-coupled receptor kinase 2-induced phosphorylation of 7-pass transmembrane receptor smoothened (*Smo*) via its binding to 12-pass transmembrane receptor patched. *Smo* translocation then leads to activation of *Gli* transcription factors, resulting in a higher expression of TJ proteins and a reduction in proinflammatory response of EC, leading to an improved BBB integrity [79, 80]. Further, EC-derived platelet-derived growth factor (PDGF)-B attract PCs, a main component of the BBB for its stability and functionality [81]. PC-secreted PDGF-B/PDGF-β and TGF-β reduce EC transcytosis, enhance expression of TJ proteins, and polarize astrocyte endfeet [82]. Additionally, Notch signaling through Delta-like 4 and Notch1/4 regulates angiogenesis by the precise control of vessel branching and stability during angiogenesis, which has an indirect influence on subsequent barrier differentiation [83]. These four main mechanisms result in a stable, tight, and functional NVU and the BBB. In addition to these signaling pathways, the regulated nutrient supply of parenchymal cells is highly important. For this, there are selective channels, transporters, and receptors, mediating distinct substrate trafficking across the BBB.

## Selectivity of transport across the BBB

In comparison to other selective barriers, the BBB is highly selective to handle the immense need for nutrients, allowing these to pass through while keeping pathogens out. Comparable to the BBB, the intestinal mucosa barrier also consists of epithelial cells, serving as a selective barrier for nutrients, electrolytes, and water. These intestinal epithelial cells show a similar arrangement as the endothelium at the BBB by being connected via the TJ proteins claudin and occluding and via adherent junctions. Additionally, efflux pumps like P-gp (P-glycoprotein) or ABC transporters are expressed on both [84]. But the intestinal epithelium allows a broader range of molecules to enter the lumen. Short-chain fatty acids or some polysaccharides like glucose or sucrose can passively diffuse across the epithelium or can be taken up enzymatically [85, 86]. Monoamines like dopamine, serotonin, or norepinephrine show a higher polarity at physiological pH, preventing them to enter the brain. At the intestinal epithelium, specific transporters are able to mediate the uptake of these monoamines, but the precursor proteins are able to passively diffuse across the barrier [87–91]. This helps to separate the central and peripheral monoamine system.

At the BBB, only some small lipid-soluble molecules and essential water-soluble molecules can passively diffuse across the BBB, depending on their molecular weight, their solubility, and their ionization, allowing especially hydrophobic and non-polar molecules up to around 600 Da to pass the BBB [64, 92, 93]. All other molecules, especially proteins, are closely regulated by specific channels, transporters, or receptors. This includes active efflux via e.g. ATP-binding cassette (ABC) transporters or carrier-mediated transport via solute carrier (SLC) transporters [94–96]. But often, there is not only one possibility for substrates to overcome the BBB. Some substrates, for example some P-gp substrates like the artificial molecule rhodamine, can not only be actively transported, but are also able to passively diffuse across the barrier [97].

For appropriate signaling in the brain, ion levels are controlled by specific pumps or transporters. To maintain high Na^+^ and low K^+^ concentrations in the brain, the abluminal Na^+^-K^+^-ATPase transports Na^+^ into the brain and K^+^ out of the brain. For the transfer of Na^+^, K^+^, and 2 Cl^-^ from the blood to the endothelium, cotransporters at the luminal site or calcium transporter (Na^+^-Ca^2+^ exchanger) as well as voltage-gated K^+^ channel regulate the ion transport across the BBB [64, 98].

Additionally, peripheral and neuronal amino acids are separated to avoid crosstalk and unspecific changes through neurotransmitter signaling between the brain and the blood. The specific transfer of neurotransmitters between the brain and the blood is mainly managed by Na^+^-coupled and Na^+^-independent amino acid transporters [64, 98].

Besides specific ion transporters, diffusion plays an important role in supplying the brain parenchyma. Small hydrophilic agents can pass the barrier by paracellular diffusion, limited in size due to the tight junctions. Small lipophilic molecules as oxygen, ethanol, or steroid hormones overcome the barrier by transcellular diffusion through the ECs [99–102]. Additionally, some transporters can facilitate the transport of those substrates into the brain. For oxygen, aquaporins enable a higher permeability [103]. For steroid hormones, P-gp or ABCG2 are known efflux transporters [104]. In general, for those lipophilic molecules, a combination of diffusion and active transport is seen. Further, cationic molecules pass the barrier via adsorptive transcytosis and small molecules like amino acids or glucose enter the endothelium by carrier-mediated transport [99]. The latter includes glucose transporters, L-type amino acid transporter 1, monocarboxylate transporter or organic anion-transporting polypeptide [105]. The most important selective pathway is transcytosis by specific receptors, binding distinct molecules mediating the internalization or excretion of substrates into or from the brain.

### Receptor-mediated transcytosis

Compared to the passive diffusion of small molecules, there is a lack of deeper insights into the transport mechanism of larger molecules across the BBB. But what is known is, that transcytosis displays thereby the key transport pathway for peptides, ligands, or antibodies [8]. The mechanism of the receptor-mediated transcytosis (RMT) is based on ligand binding to specific receptors, which subsequently leads to the endocytosis of this receptor-ligand complex and the following translocation to the basolateral site of the EC [105, 106]. Depending on the receptor and the substrate, RMT is directed either from the luminal to the abluminal site, thus the substrate is released into the brain parenchyma, or from the abluminal to the luminal site for transport of, e.g., waste products out of the brain into the blood stream for systemic clearance. The two most prominent receptors, which facilitate this active transport across the barrier, are the transferrin receptor 1 (TfR1) and the low-density lipoprotein receptor-related protein 1 (LRP1).

**Fig. 2:**
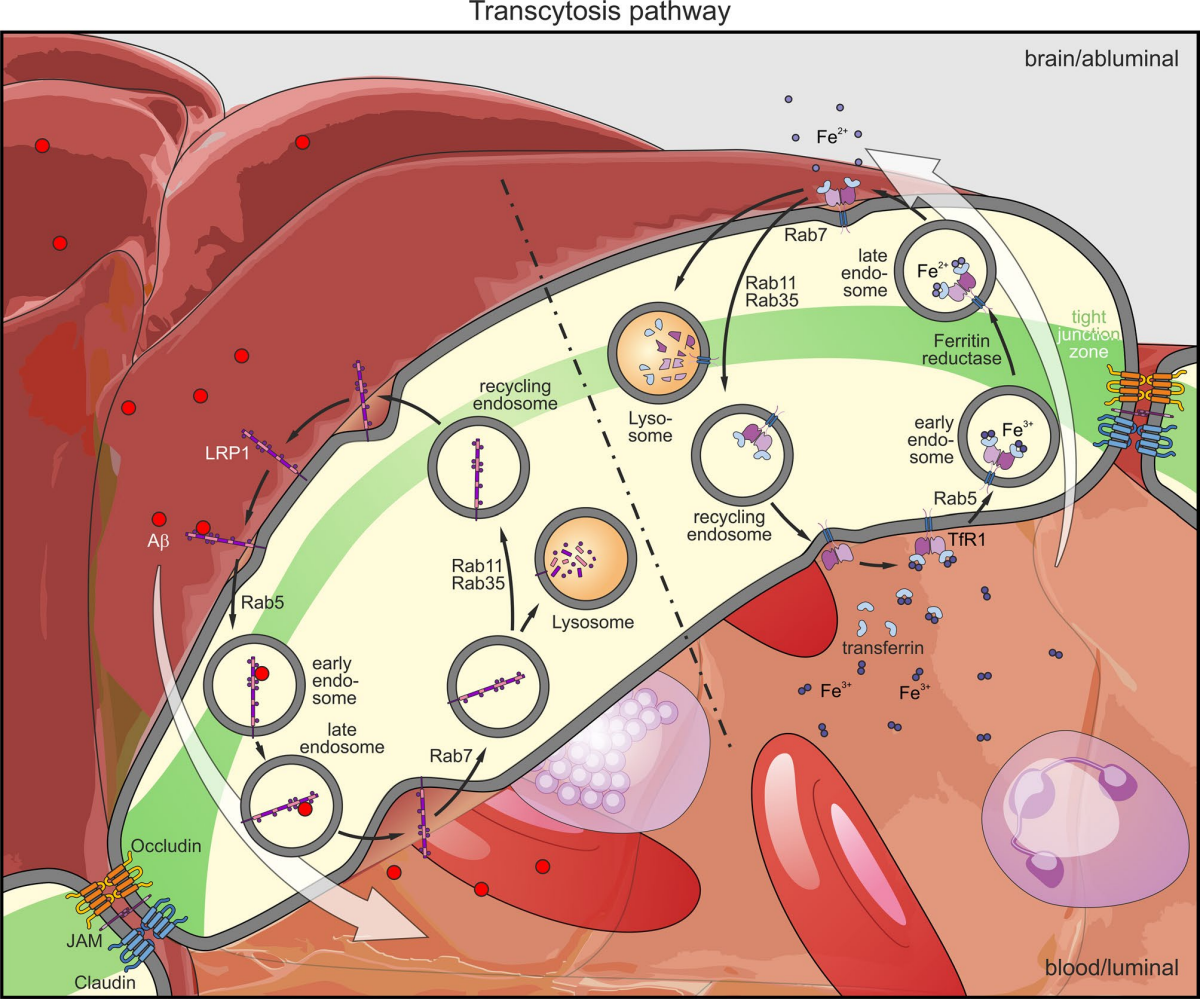
Pathway of transcytosis in endothelial cells. **Left**: LRP1-mediated transcytosis from the brain to the blood. Aβ attaches to LRP1 at the abluminal site. Endocytosis, transport, and release of LRP1-bound Aβ is, among others, mediated by Rab5. Endosomes containing free LRP1 are then either recycled back to the abluminal site via Rab11 and Rab35 or fused to lysosomes for LRP1 degradation. **Right**: TfR1-mediated transcytosis from the blood to the brain. Fe^3+^ attached to Tf binds TfR1. The complex is subsequently internalized and transported to the basolateral site. During transcytosis, Fe^3+^ is reduced by the ferritin reductase and released as Fe^2+^ into the brain parenchyma. The Tf-TfR1 complex is then either degraded by lysosomal fusion or recycled back to the luminal site

TfR1 is expressed on nearly every cell type, being responsible for transferrin endocytosis and subsequent iron supply of the cells [107]. The TfR is a homodimeric protein, connected via helical domains by two disulfide bonds at residues Cys89 and Cys98 [108, 109]. The protease-like domain and the apical domain are ectodomains of the receptor, facing the extracellular space [108]. A single transmembrane domain anchors the TfR1 in the plasma membrane and the short 5 kDa cytoplasmic domain remains for intracellular phosphorylation signals for its biological functionality [109]. The expression of TfR1 is regulated by iron-regulatory proteins (IRPs) and iron-response elements (IREs) [110]. The hairpin-structured proteins IRP1 and IRP2 are mainly involved in this iron-dependent regulatory process. IRP1 is thought to bind the IRE of TfR for an upregulation of TfR expression and further, under iron-rich conditions, is able to bind the 5´-region of ferritin mRNA to inhibit its translation [111–113]. IRP2 is ubiquitinated by plentiful iron stores which lead to its degradation whereas an iron-poor environment results in an IRP2 upregulation favoring TfR1 expression [114, 115]. In general, there are two main forms of TfR, TfR1 and TfR2, whereby TfR1 plays a more prominent role in iron homeostasis due to its ubiquitous expression and its 25-fold higher affinity to transferrin (Tf) [116]. For iron uptake and regulation, Tf, which contains two homologous domains (“N and C lope”), binds Fe^3+^, leading to a conformational change of Tf [117]. This loaded Tf binds the TfR1 with higher affinity than the mono- or apo-transferrin [118]. The Tf/TfR1 complex is then internalized via clathrin-coated pits, and iron is reduced and released in the acidic endosomal environment. Subsequently, TfR1 is recycled back to the membrane and Tf is released from the TfR1 to be available for the next cycle [119] (Fig. 2). For TfR1, endocytosis was seen at the apical as well as at the basolateral site of the plasma membrane of polarized cells [120]. In endothelial cells, TfR1 is mostly expressed on the luminal site, providing a favorable localization for drug delivery approaches into the brain [9].

In contrast, LRP1 is mainly expressed at the abluminal site of the endothelial cells and belongs to the low-density lipoprotein receptor (LDLR) family. It is a 600 kDa protein, divided into an alpha chain, which contains the extracellular N-terminal ligand-binding subunit, and a beta chain, marking the intracellular membrane anchored part, which is necessary for adaptor protein binding, signaling and endocytosis [121, 122]. Primary targets for LDLRs are low-density lipoproteins (LDLs). Further, other lipoprotein particles as apoE or very-low-, intermediate-, or high-density lipoproteins show an affinity for LDLR [123]. Due to the binding of lipoprotein particles and the nuclear regulation of cholesterol, LDLRs play a crucial role in cholesterol homeostasis, cardiovascular diseases, and atherosclerosis [105, 124]. Besides these diseases, apoE endocytosis via LDLR influences the accumulation and clearance of amyloid β peptide (Aβ), which is one of the main pathological hallmarks of AD [125]. The transmembrane protein amyloid precursor protein (APP) is normally cleaved by an α-secretase, e.g. a disintegrin and metalloproteinase with thrombospondin motifs (ADAM) 10 or 17, resulting in soluble APP (APPsα) and the C-terminal fragment C83 [126, 127]. Followed by a γ-secretase cleavage, non-amyloidogenic peptide is generated. This pathway of APP cleavage is therefore called the non-amyloidogenic pathway [128]. Especially in familiar mutations (FAD) in the *APP* gene occur, leading to a favored cleavage by β-secretases such as beta-site amyloid precursor protein cleaving enzyme 1 (BACE1), resulting in soluble APP (APPsβ) and the C-terminal C99 fragment [129, 130]. Further cleavage by γ-secretases results in the generation of Aβ. Thus, this pathway is also known as the amyloidogenic pathway [128]. Depending on this cleavage, Aβ can contain between 39 to 43 amino acids [131, 132]. While Aβ_39_ is non-toxic, Aβ_40_ and especially Aβ_42_ can cause the accumulation of Aβ peptide in form of monomers, soluble oligomers, protofibrils, and fibers [133]. In the brain, Aβ is eliminated by enzymatic degradation, phagocytosis by microglia or astrocytes, or transported across the BBB to the peripheral organs for a systemic clearance [134]. At the BBB, one of the most important transporters for Aβ clearance is LRP1 [135]. Associated with phosphatidylinositol binding clathrin assembly (PICALM) protein, LRP1 internalizes Aβ via a clathrin-mediated endocytosis. PICALM stabilizes the LRP1- Aβ complex and directs it to the early endosome and sorting endosome for Aβ transcytosis and lysosomal degradation [136, 137] (Fig. 2). Carrying this important regulatory function, PICALM is the second highest genetic risk factor for AD after apoE [138]. Additionally, P-gp seems to be involved in this transcytosis cassette [10]. In the interstitial fluid, Aβ can form complexes with apoE2, 3, or 4. Aβ-apoE2 or 3 complexes interact with LRP1, resulting in endocytosis and clearance to the blood. Aβ-apoE4 inhibits LRP1-mediated endocytosis and is therefore less degraded, resulting in Aβ accumulation in the brain, making apoE4 allele another major risk factor of AD [137, 139].

The internalization of substrates via LDLR family members is based on the clathrin-dependent pathway by formation of endosomes. The lipid dissociates from the receptor due to lysosomal hydrolysis, where the pH in the vesicle decreases, and the receptor is recycled back to the cell surface [124]. In addition, P-gp is a luminally expressed efflux transporter, mediating the waste clearance and detoxification from the brain and inhibiting the internalization of xenobiotics or drugs [140]. Studies could show that P-gp not only contributes to the Aβ clearance, but also is co-localized with LRP1, indicating its role in transcellular transport from the abluminal to the luminal site [10, 94, 141].

In contrast to the hypothesis that LRP1 and P-gp are relevant for Aβ transcytosis, Nazer et al. revealed in their study a lack of Aβ transcytosis in MDCK cells. P-gp upregulation could thereby not promote Aβ transport. Additionally, LRP1 activity only led to an uptake and degradation of Aβ, but did not result in transcytosis of the peptide [142]. As MDCK cells are epithelial cells, they do not serve as good model for endothelial transport studies. Ito et al. investigated the BBB clearance of [^125^I]-labeled human Aβ_40_ in vivo. They demonstrated, that LRP1 blocking did not alter the Aβ clearance, but still stopped the elimination of the LRP1 substrate α2 M. Therefore, they referred to the hypothesis, that LRP1 might not be one of the main clearance transporters for Aβ as additional pathways are present, mediating Aβ elimination from the brain [143]. Supporting this, Candela et al. found out that LRP1 on endothelial cells is not involved in Aβ transport across an in vitro BBB [144]. This was also proven by LRP1 inhibition and subsequent investigation of Aβ and LRP1-substrate (tPa) transcytosis. Hereby, as also shown by Ito et al., only a reduction in eliminated LRP1-substrate was demonstrated, but not an effect of LRP1 blocking on Aβ transport. But additionally, they could demonstrate that LRP1 expression is also associated with the presence of pericytes, showing the importance of intracellular assembly and their role in BBB functionality. Other studies, which used endothelial cells for the LRP1-mediated transcytosis of Aβ could demonstrate the LRP1-dependent transport of Aβ across such cells [10, 135, 145]. These controversies show the discrepancies in LRP1 studies and its role in Aβ clearance, highlighting the still existing gap in research for characterization of LRP1-mediated transcytosis.

### Transcytosis pathway

Intracellular trafficking is mainly mediated by Ras-associated binding (Rab) GTPases [146]. They are involved in both endocytosis as well as exocytosis of substrates or receptors [147] (Fig. 2). For this mediation, Rab guanine nucleotide exchange factors and GTPase activating proteins regulate the shift between the inactive Rab variant (guanosine-5’-diphosphate (GDP)-bound) and the active Rab variant (guanosine-5’-triphosphate (GTP)-bound) [147, 148]. In their active form, Rab proteins interact with different effectors which have various functions including cargo sorting complexes, motor proteins for vesicular transport, or complexes which manage the fusion of vesicles with the acceptor membrane [146, 147, 149]. Within the cell, Rab proteins occur in different cell compartments including endoplasmic reticulum, intermediate compartment, trans-Golgi-network, synaptic vesicles, early endosome, late endosomes, endolysosomes, and lysosomes [150, 151]. Rab 5 is mainly associated with the endolysosomal system since a loss of Rab5 is associated with less endosomes and lysosomes [152]. While Rab9 is an autophagosomal marker, Rab7 is found on late endosomes and lysosomes, and Rab11 plays a central role in endocytosis and recycling of receptors via a Ca^2±^induced exocytosis, especially for TfR1 [151, 153–155]. Rab35 shows various functions including vesicle trafficking, endocytic recycling, or exocytosis [156]. Besides the function of vesicular transcytosis, Rab13 is important for the formation of TJs among epithelial cells [155].

As mentioned before, this transcytosis is responsible not only for the uptake of substrates into the brain parenchyma, but also for the secretion of waste products out of the brain. This direction is similarly closely regulated by different clearance systems.

### Waste clearance at the BBB

To control the protein content in the brain, unneeded plasma proteins are mainly removed from the brain via the choroid plexus [33]. The combination of specific uptake and exocytosis as well as the clearance of waste from the brain is important to keep the brain homeostasis in physiological conditions. Disruption of this homeostatic system leads to neurological diseases and, depending on the impairment of the BBB, to neurodegeneration and subsequent cognitive decline and neuroinflammation.

Waste products from neural cells can cause severe damage within the brain. Often, failure in waste clearance is accompanied by neurological diseases such as AD. An accumulation of tau protein or Aβ leads to the pathological phenotype of the disease [157]. The BBB limits the exchange of molecules between the brain and the blood. For eliminating waste products, there are two main clearance systems: the CSF within the brain parenchyma (the “glymphatic pathway”), and the vascular system across the BBB [7]. Small molecules like ions or lactate are transported across the BBB to be systemically cleared through the blood stream [158]. Some proteins such as Aβ, insulin, or transferrin have specific receptors at the BBB, managing the systemic clearance [159–161]. Larger molecules like sucrose or inulin, which do not have specific receptors for a BBB-mediated clearance, exit the brain parenchyma via the perivascular, glymphatic system or perineural routes [158].

The CSF, located in the cerebral ventricles and subarachnoid space, not only protects the brain against injury by displaying a buffering system around the brain parenchyma, but also maintains brain homeostasis by its clearing capacity [162]. At the cerebral aqueduct, in the ventricles, and the subarachnoid space it is therefore able to remove high molecular weight waste products out of the brain [158, 163]. Besides the CSF, ISF, extracellular fluid surrounding the cells in the brain parenchyma, is responsible for a fast delivery of nutrients to the cells and a rapid removal of waste products [158, 164]. Some waste products of cells can also be a substrate for other cells. Neurons and other cell types in the brain are able to use lactic acid, a waste product of astrocytes, for their synthesis of acetyl-CoA [165]. Additionally, neurotransmitter levels in the brain parenchyma must be controlled by a homeostasis of storage and clearance to avoid impaired neuronal signaling [158].

The vascular clearance of waste products across the BBB comes in two different variants: The passive pathway, which is further divided into the paracellular and the transcellular diffusion, and the active transport through transporters or receptors. Due to the TJs between the ECs, paracellular diffusion is very restricted to small solutes [158]. Small waste products like aqueous compounds, ethanol, methanol, glycerol, and urea can be transcellularly released into the blood vessels [137]. Larger molecules or proteins need specific transporters or receptors to exit the brain. The most prominent transporters are different ATP-binding cassette (ABC) transporters, including ABCA1 and ABCG2 for steroids or ABCC4 for metabolite efflux, and solute carrier (SLC) transporters., including GLUT1 for glucose transport, LAT1 for amino acids, MCT1 for lactate, pyruvate or ketone bodies, or OAT3 as a waste shuttle [137, 166–170]. The latter are well known for transporting waste products from the brain parenchyma to the ECs and subsequently into the bloodstream [137]. Through this RMT, large molecules are actively transported across the BBB. This includes the Aβ clearance by LRP1, insulin transport through the insulin receptor, or transferrin transport by TfR1 [125, 160, 161]. LRP1 binds Aβ in an apoE-dependent manner, leading to Aβ efflux. ApoE is mainly responsible for the lipid homeostasis and lipid uptake, and Aβ. Aβ accumulation increases by E2 < E3<E4 variants and Aβ clearance decreases by E2 > E3>E4 variants, making the *apoE4* gene to one major genetic risk factor in AD [171]. In AD brains, apoE4 levels are increased, leading to a destabilization of the BBB, an impaired homeostasis, and a reduced Aβ clearance due to the reduced affinity of apoE4 to Aβ [140, 172].

Many neuropathological diseases are also associated with disturbances in waste clearance. After traumatic brain injuries or strokes, the BBB can be disrupted by losing TJ proteins or a lower expression of transporters such as LRP1 [36, 140, 173]. Further, the intracranial milieu can be disturbed, causing neuroinflammation [140]. Oxidative stress, caused by reactive oxygen species (ROS), can lead to cellular damage, modulation of TJ proteins, cytoskeleton reorganization, neuroinflammation, activation of proteases, and mitochondrial failure [174]. Also, LRP1 oxidation, leading to an increased Aβ deposition in the brain, was observed [175]. Not only is the clearance system disturbed by neurological diseases, but the general composition or signalling in the brain or at the BBB can also be affected.

## BBB impairment in age and disease

The disruption of the BBB can rely on various aspects including TJ protein expression, transporter or receptor expression or the altered assembly of the NVU. Well-known molecules which contribute to the pathological BBB breakdown are vasoactive proteins (e.g. VEGF), reactive oxygen species (ROS), inflammatory cytokines as interleukin (IL)-1, IL-6, or tumor necrosis factor (TNF)-α, proteases (matrix metalloproteinases MMP2 and MMP9), and leukocyte adhesion molecules (P-selectin, E-selectin, intercellular adhesion molecule (ICAM), vascular cell adhesion molecule (VCAM)) [8]. VEGF is an angiogenic factor increasing vascular permeability [176]. Further, it causes a decrease in the TEER, representing a higher permeability of the BBB, and decreased claudin-5 and occludin levels in endothelial cell culture [177]. In vitro, ROS showed reorganization and loss of TJ proteins through RhoA, PI3 kinase, and PKB/Akt [178]. Also, free radicals lead to a decrease in TEER in the frog brain venular endothelium [179]. TNF-α caused a BBB opening in vivo after intracranial injection and a loss of VE-cadherin and subsequent gaps in the endothelial cells [180, 181]. Furthermore, NF-κB is activated by TNF-α causing an internalization of TJs [182]. IL-1β causes systemic inflammation and BBB disruption [183]. MMPs seem to cleave TJ proteins, thus leading to BBB leakage [184]. One study suggests that MMP9 is secreted from invading leukocytes and is upregulated by VEGF, ROS, and inflammatory cytokines [185, 186]. An increase in all those different factors can cause neurological decline and neuropathologic symptoms in age and disease. But besides changes in protein expressions, also the impact of the gut microbiota on BBB functionality is discussed. There are studies revealing correlations between serum bile acid levels and CSF Aβ and tau [187]. This was strengthened by revealing neuroprotective functions of Takeda G protein receptor 5, a key receptor for bile acids [188]. But causal evidence for a connection between disturbed gut microbiome and neurodegeneration in human remains unclear.

With age, the vascular system is weakened, resulting in changes in blood pressure and further in potential damage to the BBB [189]. One main problem in age is the reduction of TJ proteins such as claudin-5, occludin, or ZO-1 at the ECs [6]. Some molecules, whose expressions seem to be enhanced during aging, were shown to impact the expression of TJ proteins. These include sirtuin-1, miR-195, or angiotensin-II [190–192]. Until now, the exact pathway of TJ degradation remains unknown, but this breakdown leads to an increased permeability, resulting in the infiltration of the brain by, e.g., immune cells or pathogens, resulting in neuroinflammation. But in addition to an increase in leakiness of the BBB due to a reduction in TJ proteins, a global age-related change of a shift in transport could be seen previously. Thereby, this includes a shift from ligand-specific RMT towards unspecific calveolar transcytosis in brain ECs, which was demonstrated, among others, for Tf RMT [193].

Besides an impaired expression of TJ proteins, the expression of receptors or channels, which mediate transcytosis at the BBB, can be influenced during age. Also here, there is a lack of studies which investigate the changes in receptor expression. For IGF1R and NF-E2-related factor 2, potential alterations during aging have been found [194, 195]. ICAM-1 and VCAM-1 are well studied in age, showing an up-regulated expression, leading to increased T-cell invasion and vascular immune cell interaction [196–198]. What could also be shown so far is a reduction of P-gp efflux capacity not only in AD patients but also during age of healthy individuals [199, 200]. This could enhance the accumulation of toxic substrates and an impairment of the transcytosis at the endothelium, favoring a neurodegenerative phenotype during age. Recent proteome studies revealed the change of the expression of various transporters. Including ABCB1, ABCG2, ABCB11, ABCC4, and the SLC proteins SLC22A3, SLC22A6, SLC29A1, SLCO1A2, and SLCO2B1, a reduced expression could be detected [201]. Whereas the ABC transporters are mostly responsible for the efflux of waste products or metabolites, the SLC proteins mediate the uptake of molecules. A reduced expression of these proteins subsequently may lead to a dysregulation of the BBB homeostasis in age, also favoring a neurodegenerative pathology.

Additionally, cellular hallmarks in age can be seen. Astrocytes show a decrease in number and size during aging [202]. Due to this, the surface covered by astrocyte endfeet is reduced, leading to a disruption of the NVU [203]. Further, the expression of astrocytes is shifted more towards an A1-like reactive phenotype. Here, the NF-κB pathway as well as the TGFβ and the complement pathway are enhanced, leading to the secretion of inflammatory factors causing neuroinflammation and subsequent degradation of neural cells and therefore an impaired signaling within the NVU [204–208]. Also the glycolysis of astrocytes, which is regulated by noradrenaline secreted by neurons, seems to be inhibited due to a decline in noradrenaline levels, resulting in a reduced ATP supply for neurons [209, 210]. The higher release of ROS in astrocytes can, among others, lead to reduced expression of IGF1R, leading to a lower rate of gluconeogenesis [211, 212]. Immune cells of the CNS, such as perivascular macrophages or microglia show an increase in expression of several surface molecules, causing the activation of other immune cells or the release of neuroinflammatory factors [213]. Especially on microglia, toll-like receptors are upregulated, displaying one of the key receptors for lipopolysaccharide (LPS) and an initiator of NF-κB inflammatory pathway [214]. Also, endothelial dysfunction by a reduced nitric oxide bioavailability, increased endothelin-1 production, and more oxidative stress is seen during age [215]. To sum up, the altered expression of proteins and the subsequent neuroinflammation, impaired signaling, or downregulated metabolism of NVU cells are mainly seen during age, leading to neurodegeneration and BBB impairment.

Besides ageing, different neurological diseases can cause similar phenotypes, leading to BBB disruption and causing subsequent severe cognitive or motoric impairments. During and after a stroke, the BBB becomes more permeable, resulting in an influx of plasma molecules and macrophages or neutrophils [216]. Also, multiple sclerosis (MS) is characterized by lesions, leading to BBB disruption and subsequent influx of molecules and blood-derived immune cells, which are then trapped in the brain, leading to neuroinflammation [5, 217]. Additionally, studies have shown evidence that in MS, TJ proteins are reduced, resulting in a more permeable BBB [218]. Besides this, there are neurodegenerative disorders, which are characterized by BBB impairment. ALS pathogenesis is based on the loss of motor neurons in the spinal cord and the motor cortex. Studies showed that in mice ALS models, BBB leakage, a reduction of endothelial GLUT1, and reduced level of TJ proteins like claudin 5, ZO-1, and occludin occurred [219, 220]. PD is mainly characterized by a loss of dopaminergic neurons in the midbrain, as well as an increase in the density of endothelial nuclei and polymorphisms in P-gp are observed, which might indicate abnormal vasculature regulation at the BBB [221, 222].

Several studies have reported early BBB breakdown in individuals with mild cognitive impairment or in aging populations prior to extensive neurodegeneration, suggesting that vascular dysfunction may represent an early event in disease development. AD is one of the most common form of dementia (60–80%), characterized by progressive cognitive decline and memory impairment [223]. It is estimated that until 2050, the patients suffering from AD will increase up to 140 million [224]. Divided into a sporadic and familial form (FAD), the sporadic form shows a late onset, whereas FAD as an autosomal disorder is known for an earlier onset [225, 226]. In both, accumulation of Aβ in the brain parenchyma as so-called Aβ plaques is driving the pathogenic phenotype of the disease [225]. This accumulation is caused by an impairment of the BBB, mainly based on the clearance pathway by apoE and LRP1, resulting in less Aβ clearance through the endothelium [4]. Thereby it is shown that in 9-month-old mice, the expression levels of vascular LRP1 decreased compared to 2-month-old mice. This was also seen in human AD brains. Hence, a correlation between vascular LRP1 downregulation and an Aβ accumulation was identified [159]. Besides a change in LRP1 expression and an impaired Aβ clearance, LRP1 deletion in various brain-associated cell types like ECs showed an increase of brain Aβ. This was explained by a reduced APP internalization through LRP1 and Fe65, resulting in a decrease in intracellular APP cleavage. Intracellular cleavage thereby favors β- and γ-secretase cleavage, leading to more Aβ production [227]. But there is not one general LRP1 dysregulation, which leads to AD pathology. Studies also revealed that changes in LRP1 expression are cell type specific. Vascular LRP1 seems to be upregulated and, thus, leading to an increased Aβ burden, while neuronal LRP1 might be downregulated, resulting in higher synaptic Aβ toxicity and tau aggregation [228]. As another major LRP1 variant, soluble sLRP1 levels in the plasma demonstrate a significant decline as well as hepatic LRP1, showing an altered systemic Aβ clearance [229, 230].

Contrary to this, single-cell-RNA sequencing data revealed nearly no detectable expression of LRP1 in endothelial cells [231]. That would contradict all previous studies demonstrating LRP1 expression on ECs and LRP1-mediated transcytosis. But as these data only reveals RNA levels within the cells and not protein levels, no deductions can be drawn regarding transcytosis. Another study demonstrated, that hippocampal LRP1 knock-down in APPswe/PS1dE9 mice did not alter the Aβ40/42 ratio, thus, showing no change in amyloid deposition [232]. The authors discussed that the lack of a complete knock-down or the overtaking of LRP1 family members could have led to these results. But in this study, a knock-down of LRP1 in microglia was not shown so far, which could also explain further Aβ clearance and the unchanged Aβ burden in the hippocampus.

Further, it was demonstrated that in the cortex of 5x FAD mice, the TfR1 expression is upregulated, causing elevated iron levels, which was already seen in AD [233]. Further, iron can favor Aβ deposition and tau phosphorylation, supporting the AD phenotype. These changes in TfR1 expression also seem to be cell specific. While an HIF-1-dependent upregulation in cortical tissue was seen, no changes could be observed on brain microvessels of 5x FAD mice. Contrastingly, whole homogenates from human post-mortem parietal cortex and hippocampus did not reveal a change in TfR1 levels between AD patients and healthy individuals. Similarly, isolated human or mice brain microvessels did not demonstrate significant differences in TfR1 protein levels between control and AD cohort [234]. Additionally, in an in vitro model of human immortalized brain ECs, Aβ pathology did not affect TfR1 expression, depicting the arising discussion about its effect of neurodegenerative diseases on the BBB but also highlighting the benefits of TfR1 targeting for drug delivery due to its consistent expression [233].

However, several studies suggest that BBB dysfunction may arise as a consequence rather than a primary cause of neurodegeneration. Accumulation of Aβ has been shown to impair EC function and compromise TJ integrity, thereby contributing to secondary disruption of the barrier. In addition, inflammatory processes and oxidative stress associated with Alzheimer’s pathology may further aggravate vascular damage and BBB permeability [23]. In this context, BBB impairment is increasingly considered part of a dynamic and reciprocal interaction between vascular dysfunction and neurodegenerative processes, rather than a single initiating event in the development of AD. But it still remains unclear, if the changes of BBB properties appear consistently, or what precisely determines the various alterations or what they are based on.

There are also many reviews, which critically discuss the interpretation of data, which might prove the BBB disruption as a consequence of age or neurodegenerative diseases. Calon thereby refers to methodological issues and subsequent misinterpretation of variables, which might be often unrelated to the BBB [235]. His review emphasizes the necessarily critical use of the terms BBB “disruption”, “permeabilization”, or “opening”, as these oversimplify the complexity of effects on the BBB. Additionally, he wants to draw attention on the methodological bias which can arise when the detected biological effects are described by using larger molecules instead of small compounds for characterizing BBB functionality.

But what is clearly worth to emphasize is that there is a high need for treatment options especially for neurodegenerative diseases, various promising approaches were shown to either help existing drugs overcome the BBB or modifying drugs in the way that they can enter the brain parenchyma.

## Approaches for drug delivery and transport modification into the brain parenchyma

As described before, the BBB displays a highly complex and selective barrier. This property makes delivery of drugs into the brain complicated. Direct intrathecal administration via lumbar puncture or ventricular injections have been previously performed, but the incidence for side effects is high [236, 237]. These include an increase in intracerebral pressure, seizure, encephalitis, meningitis, and other infections [238]. Besides this, the distribution in the brain is mostly limited to the CSF which does not circulate in the entire CNS [238]. In 1975, Sinkula and Yalkowsky reviewed the first approaches to use “prodrugs” [239]. These drugs are modified in a way that their uptake becomes more specific and more effective [239]. The most modifications include physicochemical modifications to increase the absorption and distribution of the drug [239]. But mostly, there was no specific targeting of the drug to distinct regions in the body, causing again side effects [239]. Current approaches aim to efficiently target certain regions or even cell types and to reduce the rate of unspecific drug delivery, thus trying also to reduce upcoming severe side effects. The focus for drug delivery across the BBB is on directly modulating or targeting receptors at the endothelium of the brain. Besides the chemical modification of the drug itself to make it a target for the distinct receptor, antibodies which directly target the receptor are the basis of today’s approaches [240–243]. Targeting receptors on endothelial cells leads to endocytosis and transcytosis of the receptor including the substrate [244]. The most common target is TfR1. Especially in AD, targeting TfR1 seems to be a promising candidate. In preclinical studies, bispecific antibodies against TfR1 and either Aβ or BACE1 presented a higher uptake of the antibody into the CNS [11, 12, 245–248]. Besides antibodies for treatment options, the use of nanoparticles, nucleotide-based approaches, or the application of ultrasound for an improved antibody uptake are in the focus of today’s research.

### Ultrasound-based antibody delivery

**Fig. 3:**
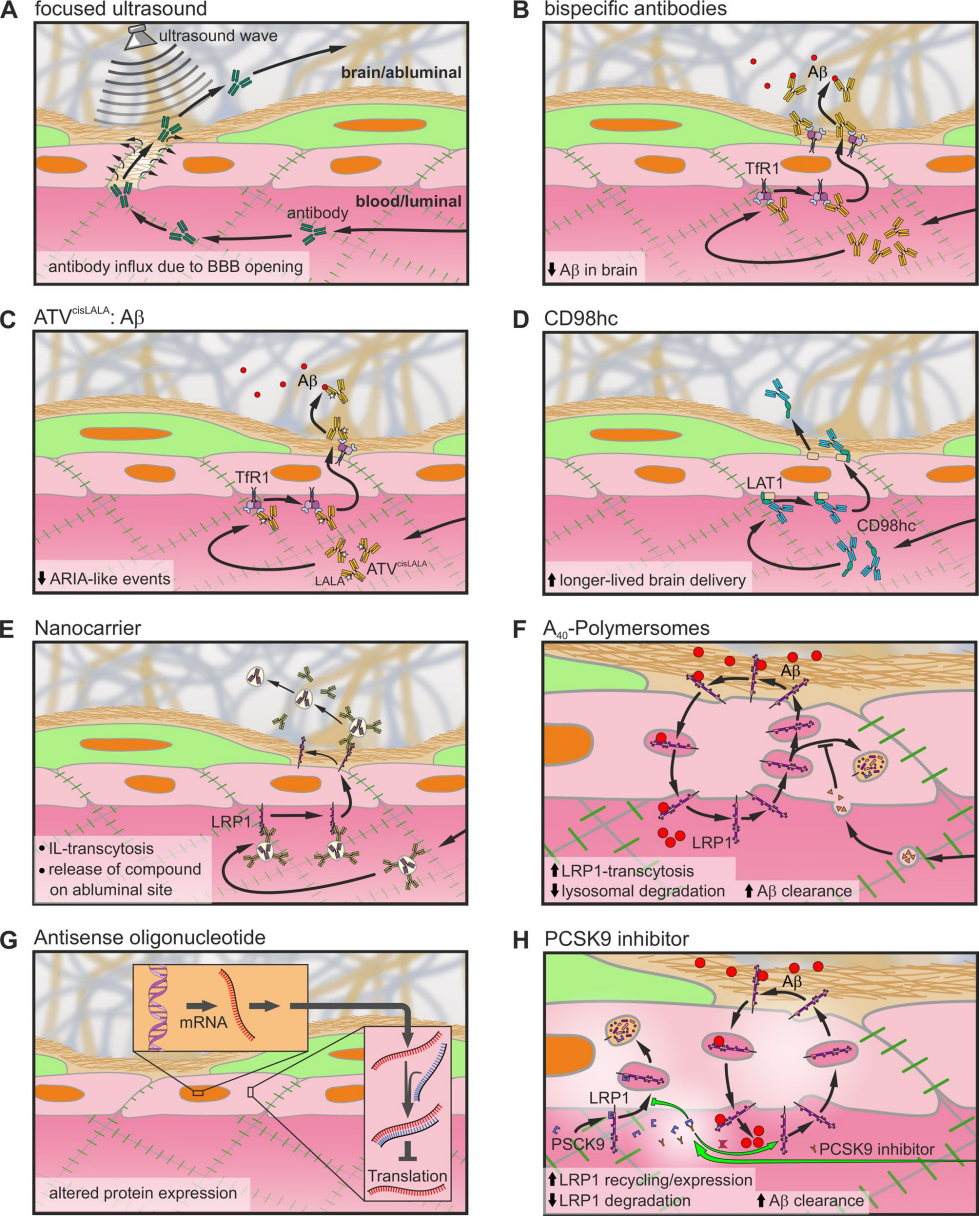
Overview on the hypothetical mechanisms of drug delivery and transport modification methods. (**A**) Focused ultrasound to open the BBB for more antibody penetration. (**B**) Bispecific antibodies targeting TfR1 and BACE1 to reduce Aβ production. (**C**) ATV^cisLALA^:Aβ reduces cell cytotoxicity and ARIA-like events. (**D**) CD98hc modification of antibodies achieves a longer-lived brain delivery. (**E**) Nanocarrier, e.g. immunoliposomes (IL) incorporating antibodies or compounds, which are shuttles across the BBB by attaching to transcytosis-mediating receptors. (**F**) A_40_-polymerosomes enable more LRP1-mediated transcytosis and subsequent more Aβ clearance, while reducing LRP1 lysosomal degradation. (**G**) Antisense oligonucleotides can influence the receptor expression on the cell surface. (**H**) Inhibition of PCSK9 to increase LRP1 expression on the cell surface and subsequent Aβ clearance

Via ultrasound, the BBB can be opened for a short period of time to allow subsequent given drugs to enter the brain [151] (Fig. 3A). There are two approaches of ultrasound: Focused ultrasound (FUS) and low-intensity pulsed ultrasound. FUS is not tested in details regarding a potential therapeutic approach for drug delivery [249]. There are first approaches to treat AD patients with an anti-Aβ antibody (aducanumab) while applying ultrasound to one hemisphere. The Aβ concentrations were reduced by 32%, but the treatment was often associated with few adverse events like headache [250]. But in this study, only three participants were analyzed, so bigger cohorts are necessary to prove the effectiveness of the combined treatment. In another study, HER2-positive breast cancer metastasis in the brain were treated with trastuzumab during focused ultrasound. 20 infusions were necessary, but were well tolerated, showing the potential of this approach [251]. For low-intensity pulsed ultrasound, devices are directly implanted into the skull. It could be shown that low-intensity pulsed led to an increased uptake of drugs into the brain across the BBB. The brain/plasma ratio increased by up to 5.8-times, but deeper insights into the pharmacological effectiveness of the drug remain unknown [252]. Also in AD, patients received those low-intensity pulses leading to an increased Aβ and tau-aggregate clearance. But in this study, only nine patients were sonicated within a small area, demonstrating a lack of significant improvement of AD pathology after this treatment [253]. Despite their low side-effects, FUS and low-intensity pulsed ultrasound have to be applied every time when antibodies are given to the patient to open the BBB. This repetitive application can lead to infiltration of many other substrates and potential pathogens, causing inflammatory processes. The extent of its impact and any further consequences have not yet been fully examined. This is why the innovation of nanoparticles or antibodies which can immediately cross the BBB by, e.g., RMT is mainly the core of today’s research.

### Modified antibodies cross the BBB

The idea of using antibodies (mAbs) for amyloid plaque reduction is based on a labeling mechanism for, e.g., microglia in the brain to become activated by the mAb-coated Aβ plaques. Until now, it is not fully understood how this activation process and the subsequent clearance of the plaques is working and if only the mAb-coated plaques are phagocytosed or also the free aggregates [254]. However, it should be pointed out that the application of mAb for amyloid plaque reduction shows significant improvements for AD patients. The two most prominent mAbs for AD treatment are lecanemab and gantenerumab.

The history of the development of anti-Aβ therapies started in 1999, where Schenk et al. tried to immunize APP-expressing mice with Aβ_42_. They could demonstrate that an immunization of young animals resulted in a later AD onset and immunization of older mice with AD phenotype reduced the progression of the disease [255]. To extent these findings, the first clinical study on immunization with Aβ_42_ started, but was not continued due to severe side effects as meningoencephalitis [256]. Since 2012, different antibodies targeting Aβ fibrils, aggregated Aβ, or soluble Aβ have been investigated. Reaching from solanezumab and aducanumab, which both did not show significant clinical effects, to donanemab and lecanemab, which are the most recent and most promising antibodies [257–260].

Lecanemab (Leqembi®) is a monoclonal IgG1 antibody, targeting soluble and aggregated Aβ like protofibrils, fibrils, and plaques. The binding affinity increases over monomers, soluble fibrils, and insoluble fibrils [261–265]. An effective Aβ plaque reduction was shown in animal models with a murine variant of lecanemab before [261, 263, 266]. In a clinical trial, lecanemab was applied to patients over 18 months to verify the most effective dose (ED90), which was set to 10 mg/kg biweekly according to ADCOMS. In this double-blind, phase-2a study, a dose-dependent reduction of Aβ was seen in PET scans of those patients [265]. Also phase 3 studies could show the effectiveness of lecanemab for AD treatment, so lecanemab became approved by the European Medicines Agency (EMA) [267–269]. But the application of lecanemab must be well evaluated due to the occurrence of Amyloid-Related Imaging Abnormalities (ARIA). One study showed that 21.5% of the patients suffer from ARIA, whereas 1.1% exhibit severe symptoms due to ARIA, including hemorrhagic symptoms and edema [21, 270]. Patients with *apoE4* homozygotic gene status and patients who receive anticoagulants are not advised to take lecanemab since more and severe ARIA occurs [270–273]. Human pharmacokinetics of lecanemab need to be further evaluated. With the analog mouse antibody mAb158, only 0.3% of the injected dose/gram brain tissue, showing the low bioavailability [246].

The humanized IgG1 antibody donanemab, directed against an N-terminal pyroglutamate epitope, showed in a phase 1b study with amyloid-positive mild cognitive impairment to moderate AD a significant reduction in Aβ plaque burden. A follow-up phase 2 trial could not achieve a significant improvement, but a slight enhancement in cognition of the patients [274]. Recent studies demonstrated infusion-related reactions in most of the treated patients after some injections [275]. However, donanemab was FDA approved in July 2024, as it showed slower cognitive decline and a significant reduction in Aβ plaque load [276]. The bioavailability and pharmacokinetics of donanemab have to be further evaluated as discussed for the other antibodies.

Gantenerumab as another monoclonal anti-Aβ antibody, that, in in vitro studies, showed an increased phagocytosis of oligomeric Aβ_42_ and an improvement of long-term potentiation in rat brains [277]. As a fully human Aβ IgG1 antibody, it can promote microglial phagocytosis of aggregated Aβ as fibrils or plaques [278]. Previous in vitro studies have shown that gantenerumab shows one of the highest affinities to aggregated Aβ and soluble oligomers, which would make it a promising candidate for lowering the Aβ burden in AD patients [277]. There are ongoing phase 3 clinical studies investigating long-term safety and tolerability, but studies have shown similar ARIA events as for lecanemab [22]. Investigation of pharmacokinetics and bioavailability have to be investigated further.

To obtain higher uptake rates of the antibodies, the idea arose to target receptors at the luminal site of the BBB for facilitating their transcytosis. The main receptor, on which the focus of today’s research is based on, is TfR1. Until now it remains still unclear how this endocytosis process is working, but several studies were able to demonstrate an increased transport of antibodies or cargos in nanoparticles when they are directed against TfR1 [248, 279].

Trontinemab (RG6102) displays an antibody resulting from a fusion of the gantenerumab complementary-determining regions (CDRs) and a human/cynomolgus monkey cross reactive TfR1-binding shuttle module [11]. In non-human primates, it was well tolerated after a single dose injection of 10 mg/kg. Trontinemab reaches deeper regions of the brain as hippocampus, cerebellum, cortex, or striatum compared to gantenerumab showing the effect of an increased uptake and a wider distribution in the brain by the TfR1-coupling [11]. Clinical studies on trontinemab are ongoing to investigate the applicability for AD patients.

As a next step, TfR1-targeting antibodies were further modified on their Fab fragments by fusing the TfR1-targeting Fc domain to BACE1 and Tau Fab fragments (Fig. 3B). This bispecific antibody was able to simultaneously engage both Aβ_40_ formation and Tau spreading in vitro [12]. In vivo, an antibody transporter vehicle (ATV) ATV:BACE1 achieved 10–40-fold higher IgG brain concentrations compared to normal anti-BACE1 antibodies. Besides the higher total amount of IgG in the brain, also the distribution, showed in cynomolgus monkeys, is significantly wider than after the application of standard mAbs [12]. The application of this ATV:BACE1 led to a 70% reduction in CSF Aβ_40_ and a 75% reduction in soluble APPβ/APPα concentration ratio in various brain regions [12]. As these results were shown in cynomolgus monkeys, this study could be a promising approach for human application. But still, the safety and immunogenicity, as well as downstream biological effects of this artificial antibody has to be further evaluated.

This approach of combining TfR1-tagreting and direct Fab-associated targeting of AD-relevant proteins was further extended to site-directed mutagenesis of the Fc fragment to generate a so-called ATV^cisLALA^:Aβ (Fig. 3C). Therefore, L234A/L235A (“LALA”) mutations are introduced in the human IgG1 (huIgG1) Fc to reduce FcγR binding [280]. Complementary to the ATV^transLALA^, the ATV^cisLALA^ reduces antibody-dependent cell cytotoxicity via the blocking of FcγR engagement. Further, no complement-dependent cytotoxicity was observed and under chronic conditions, hematologic safety was preserved in the studies, suggesting the ATV^cisLALA^ to be a potential candidate for a shuttle of antibodies into distinct tissues or cells. In 5xFAD mice, ARIA-like events were reduced by usage of the ATV^cisLALA^:Aβ compared to other amyloid therapies by anti- Aβ mAbs. But for now, the dosage and the differences in treatment of familial and sporadic AD has to be addressed in clinical studies [280].

Another form of synthetic antibodies includes the variable domain of new antigen receptors (VNARs) from single domain antibodies found in sharks. The antigen-binding paratope of VNARs contains a long CDR3, which could circumvent steric influences, leading to an increased binding of this antibody to receptors, which cannot be addressed by conventional mAbs [281]. By a phage display library, the VNAR TXB2 was identified and coupled with a human Fc (TXB2-hFc). This bivalent VNAR shuttle successfully crossed the BBB by binding to TfR1 ectodomain, appearing in brain capillaries, parenchyma, and neurons already at low dose in mice. The average molar concentration of TXB2-hFc was 13- and 21-fold higher than controls in the brain, which was not seen in other organs, showing a more specific brain-targeting of this synthetic antibody. By investigating the pharmacokinetic profile, around 3% of the injected dose were detected in the brain, which is 10-times higher than lecanemab. No competition between the TXB2-hFc and Tf or ferritin was observed and no difference between species could be detected, making it to a cross-species BBB carrier [281]. In a follow-up study, CDR3 N-terminal residues were randomized by site-directed mutagenesis to generate TXB2 variants, which display a higher TfR1 association rate and an increased brain penetration. Here, TXB4 was selected because it shows a more exclusive distribution in the brain [282]. For this approach, side-effects are not deeply characterized. Specific immune reactions in the treated brain areas have been shown but there is a lack of long-term studies to proof if this artificial antibody may be a potential candidate for human treatments.

Besides targeting TfR1, an antibody, which targets CD98hc was created. Thereby, CD98hc forms a heterodimer with LAT1 (Fig. 3D). Therefore, a CD98hc single-chain antibody was fused to the heavy chain of an IgG by addition of a flexible linker. While TfR1-based shuttles were shown to lead to a wider distribution in the brain parenchyma, CD98hc shuttles remained in close proximity or attached to blood vessels the first five days post-injection, but demonstrated a longer-lived brain delivery [283, 284]. Also, no cellular uptake of CD98hc shuttles was seen. The use and safety of this antibody modification must be further evaluated. There may be some applications where the long-term stability of the antibody could be useful, but the specific delivery to the brain parenchyma must be optimized.

### Nanocarrier as vehicles for transporting drugs into the brain parenchyma

In addition to the targeted activation of receptors with modified antibodies, nanoparticles are used to incorporate substrates and transport them across the blood-brain barrier. The development of vectorized, ligand-coupled immunoliposomes has progressed through several key milestones. Early studies introduced the concept of immunoliposomes, where antibodies are attached to liposomal surfaces to enable receptor-specific targeting of cells [285]. In 1993, Bickel et al. were able to construct a transport vector which is covered with a monoclonal anti-TfR1 antibody. The application of these vectors resulted in a pharmacological effect in the CNS of rats [286]. Subsequent work demonstrated that antibody-modified liposomes can bind to cellular receptors and undergo receptor-mediated endocytosis, establishing their potential as targeted drug-delivery systems [287]. A major advance was the application of this strategy to the BBB. Huwyler et al. showed that liposomes coupled to antibodies against the TfR1 can deliver drugs across the BBB via RMT [288]. This study provided the first demonstration that antibody-targeted liposomes could exploit BBB transport mechanisms to deliver therapeutic compounds to the brain. Later improvements focused on increasing stability and circulation time, for example by introducing PEG linkers that enhance liposome pharmacokinetics while maintaining receptor binding [289]. Finally, the use of antibody fragments such as single-chain variable fragments enabled more efficient targeting and modular design of immunoliposome systems [290].

To date, various kinds of nanoparticles have been investigated including liposomes, solid-lipid nanoparticles (SLNPs), polymeric nanoparticles, metal nanoparticles, and extracellular vesicles [13]. The properties of these vesicles must be optimized regarding their efficiency, loading capacity, cytotoxicity, and distribution. To improve specificity and transport efficiency, nanoparticles are functionalized, meaning the modification of the surface with targets for, e.g., BBB-associated receptors or transporters. There are studies showing that an apoE-functionalization of SLNPs resulted in a 3-fold increase in BBB penetration compared to non-modified SLNPs [14].

Studies expanded the approach of creating nanocarrier using alternative ligands for TfR. For example, Zhang et al. developed polymeric nanoparticles modified with the T7 peptide, a ligand that binds to the TfR1. These nanoparticles enabled the delivery of small interfering RNA (siRNA) across the BBB and into glioma cells, demonstrating enhanced brain accumulation and improved therapeutic efficacy compared to non-targeted nanoparticles [291]. One promising approach involves engineered nanobody-based carriers targeting TfR1. In a study by Esparza et al., a pH-sensitive TfR-binding nanobody was fused to macromolecular cargo to promote efficient transcytosis across the BBB in vivo. The engineered nanobody showed improved brain uptake by exploiting receptor recycling pathways and pH-dependent binding properties that enhance cargo release after crossing the EC [292]. Another strategy uses Tf-functionalized polymer nanoparticles. Gabold et al. developed Tf-modified chitosan nanoparticles designed for targeted delivery of protein therapeutics. The nanoparticles exhibited sizes of approximately 110–150 nm and showed significantly enhanced uptake into epithelial and glioblastoma cell models due to TfR1-mediated binding. The study demonstrated that increasing the density of transferrin ligands on the nanoparticle surface led to improved cellular uptake and transport efficiency [293].

Additionally, LRP1 is addressed in recent studies to be a target for drug delivery across the BBB. Although LRP1 is located on the abluminal site, it was already shown to be involved in a recycling process and to release Aβ at the luminal site, by which P-gp was seen so be involved as a luminal marker. Additional studies demonstrated the Aβ transport from the luminal to the abluminal site by LRP1 in primary pMBEC [145]. The intracellular transcytosis mechanism of LRP1, including the receptor recycling process is not fully understood yet. But as these previous results show, the idea arises that during the abluminal to luminal transport and the subsequent recycling process, LRP1 is able to bind luminally located molecules and to transport them to the brain site.

Starting in vitro, an artificial truncated form of LRP1 (mLRP1_DIV*) was used in a cell-culture based BBB model. Functionalized immunoliposomes, loaded with the γ-secretase modulator BB25 and directed against this artificial LRP1, were shown to be transported from the luminal to the abluminal site, releasing BB25 successfully at the abluminal site (Fig. 3E). This was proven by the subsequent measurement of Aβ levels. In this study it was demonstrated that the incorporation of BB25 in liposomes resulted in a higher supply of the abluminal site and therefore a 8.8-fold higher shift of the Aβ composition towards more Aβ_38_ on the abluminal site compared to the free compound [15]. As this study is based on an in vitro model with an artificial LRP1 construct, in vivo studies of this approach must be addressed further.

Recent in vivo studies in mice confirmed that the targeting of LRP1 may be a promising approach for drug delivery into the brain. So-called A_40_-PO polymersomes as multivalent LRP1-targeted nanoparticles based on angiopep-2 were injected into transgenic AD mice (Fig. 3F). This treatment resulted in a 50% reduction of Aβ in the brain and restored the BBB phenotype by upregulation of PACSIN2 and downregulation of Rab5, resulting in a higher transcytosis. Already 12 h after injection, a reduction in Aβ plaques could be observed. Furthermore, cognitive performance improved 3 days after injection and was carried for months [16]. This study highlights the importance of understanding of the transcytosis pathway and the potential of modifying this pathway to restore and improve physiological properties. However, the effect on humans, especially in pathological models, needs to be further evaluated.

### Alternative treatment options

Comparative to liposomes, exosomes such as nano-sized lipid-bilayer vesicles can carry miRNAs, mRNAs, and small proteins [294]. Previous studies have shown that stem cell-derived exosomes have anti-inflammatory and immunomodulating functions. In one study, such exosomes were given to streptozotocin-induced AD-phenotypic rats intranasally. This treatment resulted in an improved Aβ clearance in the hippocampus as well as an improvement in cognitive behaviors. Further, the expression of specific markers for neuronal plasticity like integrin β1, NMDAR1, or pPKCα increased, showing a potential regeneration of the brain after the application of those stem cell-derived exosomes [17]. Since this model is a preclinical rat model without the human clinical pathogenic hallmarks of AD, the applicability of these exosomes must be further evaluated.

Antisense oligonucleotides (ASOs) are another promising strategy for treating neurological diseases. ASOs are short, synthetic, single-strand molecules which can alter RNA and reduce, restore, or modify protein expression [151, 295] (Fig. 3G). Through lumbar puncture, ASOs enter the CSF and were shown to target the SOD1 gene for ALS or tau biomarkers in AD [296, 297]. Also, ASOs were coupled with oligonucleotide transport vehicles (OTVs), which are directed against TfR1. This combination led to an efficient distribution in the brain and to a higher RNA-knockdown when applied intrathecally in non-human primates [18]. But for this approach, no long-term studies were performed and published until now, which must be evaluated for a clinical application.

Another well-established method for drug delivery in general is the use of adeno-associated viruses (AAVs). Unmodified viruses cannot pass the BBB in a sufficient amount to use them as a vehicle. Through library screening, the VCAP-102 variant was identified to exhibit an enhanced BBB penetration. In a transwell-model, this penetration was shown to be based on alkaline phosphatase, a vascular receptor on brain capillaries which can be addressed by VCAP-102. As this vehicle shows comparable affinities (10- 30 nm) and a dissociation at low pH (pH5), the properties are similar compared to other ligands of receptors as TfR1, CD98hc, or IGF1R [298]. Since the virus displayed also lower liver transduction, this AAV variant may be a candidate for encapsulation of drugs. Further, targeting of the alkaline phosphatase could also be a promising approach for nanoparticles or antibodies to cross the BBB. As these studies were performed in rodents and primates, human studies must follow to confirm the potential therapeutic effect of this approach.

One of the most recent approaches is to achieve an allosteric targeting of proteins through transmembrane domains instead of targeting extracellular structures. Advantages here are that the binding of endogenous ligands of the targeted receptors are not influenced and a “plug-and-play” mechanism is introduced since this could be used for various lipid-based drug delivery systems. Through computational protein design and structure prediction, scientists established a peptide, which can bind to the insulin receptor (IR) transmembrane domain, the so-called IR transmembrane domain-binding peptide (ITP). The subsequent clathrin-dependent IR internalization by ITP binding is independent of insulin. ITP can be easily incorporated into the membrane of lipid nanocarrier, leading to higher stability and a more robust transport through the BBB. Also, ITP seems to induce less immunoreactivity. In an 8-month mouse-AD model, ITP-coupled nanocarriers, encapsulating siRNA for BACE1 inhibition led to a cognitive improvement [299]. Since this ITP-based modification is the fundament of new design strategies for liposomal carriers, many opportunities for various drug deliveries are given by this approach.

An alternative approach instead of utilizing RMT for a more efficient drug delivery into the brain parenchyma is the modification of the expression of existing receptors at the BBB. PCSK9 is a key regulator in cholesterol metabolism [300]. It binds to the LDLRs, leading to lysosomal degradation and subsequent higher low-density lipoprotein cholesterol (LDL-C) level [19, 301]. PCSK9 mutations represent around 1% to 2% of familial hypercholesteremia cases [302]. The inhibition of PCSK9 by FDA-approved alirocumab or evolocumab leads to an increase in LDLR level and further to higher clearance of LDL-C. Besides the effect on cholesterol levels, the Rotterdam Study showed that cholesterol-lowering medications were associated with a 50% to 70% lower risk of late-life development of AD [20, 303]. As high cholesterol levels can be one prevalent risk factor for AD, addressing this cholesterol-lowering medications for the improvement of AD pathology may be one promising strategy. Nevertheless, up to date, studies are lacking demonstrating a significant effect of lowering cholesterol levels by statins in AD. But in vivo studies were able to show that extracellular PCSK9 targets LRP1 as a LDLR-family member at the surface of endothelial cells resulting in a decreased Aβ-clearance by LRP1 [20]. The injection of alirocumab in 5xFAD mice resulted in significantly reduced Aβ levels in the brain but not in 5xFAD LRP1 knock-out mice based on the increased LRP1 expression due to PCSK9 inhibition, indicating the relationship between LRP1 and PCSK9 [20] (Fig. 3H). The reduced Aβ burden was associated with significant improvements in learning and memory. Given the low constitutive expression of LRP1 at the brain microvasculature and the absence of local PCSK9 production, studies indicate that peripherally derived PCSK9 regulates BBB LRP1 levels through endocrine or paracrine mechanisms, likely affecting multiple PCSK9-sensitive receptors. By promoting LRP1 degradation, PCSK9 disrupts neuronal cholesterol homeostasis and impairs Aβ clearance [304, 305]. As neurons increasingly depend on astrocyte-derived apoE lipoproteins for cholesterol uptake via LRP1, PCSK9 activity ultimately contributes to neuronal dysfunction and neurodegeneration. Consistently, PCSK9 reduces LRP1-dependent Aβ transport in endothelial models in vitro [305, 306]. Since PCSK9 is primarily expressed in the adult liver and targets LDL receptor family members including LRP1, it emerges as a key negative regulator of both central and peripheral Aβ clearance, underscoring the relevance of PCSK9 inhibition when aiming to enhance BBB transport processes [305, 307].

## Conclusion

The brain is one of the most vulnerable organs in the human body. To protect the brain against foreign pathogens, inflammation, or infiltration of cells and proteins, the BBB shields the brain by a complex arrangement of various cells. The cells of this NVU have various functions including the transport of substrates from the blood to the brain and vice versa, the intracranial signaling for appropriate brain function, and the maintenance of a homeostatic system by mediating clearance out of the brain. A disruption of this complex barrier in age or disease is often accompanied by neuroinflammation or neuronal decline, followed by cognitive or motor impairments. But until now, different controverse studies claim other causes of BBB disruption, starting with gut microbiome, altered protein expression, or global age-related changes in transcytosis, showing the wide range of causes for BBB impairments and subsequent resulting neurodegenerative diseases. Additionally, the studies which claim BBB disruption as a consequence of age or neurodegenerative disease have to be critically evaluated regarding the gain of their results, as the term “disruption” clearly highlights massive impairments instead of small changes, which might influence BBB properties. The pathology of AD for example is yet not fully understood. Especially the role of LRP1 in Aβ generation and clearance still gives rise to a lot of discussion. The treatment of such neurodegenerative diseases remains very challenging, since the most drugs are not able to overcome the BBB and therefore to enter the brain. In the last decades, many approaches have occurred to circumvent this problem. This includes the incorporation of the drugs into nanoparticles or liposomes, the modification and functionalization of bispecific antibodies, the usage of ASOs, viruses, or the application of substances, which enhance transcytosis receptor expression. This review represents an overview of clearance and transport mechanisms across the BBB and how these pathways can be used for drug design or modification. We depicted the current state of the art in drug transporter research, and which different approaches are conceivable in near future also for clinical trials to treat neurodegenerative diseases.

## Data Availability

Not applicable.

## Abbreviations

AAV: Adeno-associated viruses
Aβ: Amyloid β
ABC: ATP-binding cassette
AD: Alzheimer´s disease
ADAM: A disintegrin and metalloproteinase with thrombospondin motifs
AJ: Adherens junction
ALS: Amyotrophic lateral sclerosis
ApoE: Apolipoprotein E
APP: Amyloid precursor protein
AQP4: Aquaporin-4
ARIA: Amyloid-Related Imaging Abnormalities
ASO: Antisense oligonucleotide
ATV: Antibody transporter vehicle
BACE1: Beta-site amyloid precursor protein cleaving enzyme 1
BBB: Blood-brain barrier
BM: Basal membrane
CDR: Complementary-determining region
CNS: Central nervous system
CSF: Cerebrospinal fluid
EC: Endothelial cell
ED90: Effective dose 90
EMA: European Medicines Agency
FAD: Familiar Alzheimer´s disease
FUS: Focused ultrasound
GDNF: Glial cell line-derived neurotrophic factor
GDP: Guanosine-5’-diiphosphate
GTP: Guanosine-5’-triphosphate
hc: Heavy chain
ICAM: Intercellular adhesion molecule
IGF-1: Insulin-like growth factor 1
IL: Interleukin
IR: Insulin receptor
ITP: Insulin receptor transmembrane domain-binding peptide
LPS: Lipopolysaccharide
IRE: Iron-responsive element
IRP: Iron-regulatory protein
ISF: Interstitial fluid
LDL-C: Low-density lipoprotein cholesterol
LDLR: Low-density lipoprotein receptor
LRP1: Low-density lipoprotein-related receptor protein 1
MS: Multiple sclerosis
NVU: Neurovascular unit
OTV: Oligonucleotide transport vehicles
PC: Pericyte
PCKS9: Proprotein convertase subtilisin/kexin type 9
PD: Parkinson´s disease
PDGF: Platelet-derived growth factor
P-gp: P-glycoprotein
PICALM: Phosphatidylinositol binding clathrin assembly
RA: Retinoic acid
Rab: Ras-associated binding
RMT: Receptor-mediated transcytosis
ROS: Reactive oxygen species
sAPP: Soluble amyloid precursor protein
*Shh*: Sonic hedgehog
SLC: Solute carrier
SLNP: Solid-lipid nanoparticles
*Smo*: 7-pass transmembrane receptor smoothened
TEER: Transendothelial electrical resistance
Tf: Transferrin
TfR: Transferrin receptor
TJ: Tight junction
VCAM: Vascular cell adhesion molecule
